# Thermal Expansion and Other Thermodynamic Properties of *α*_2_-Ti_3_Al and *γ*-TiAl Intermetallic Phases from First Principles Methods

**DOI:** 10.3390/ma12081292

**Published:** 2019-04-19

**Authors:** David Holec, Neda Abdoshahi, Svea Mayer, Helmut Clemens

**Affiliations:** Department of Materials Science, Montanuniversität Leoben, Franz-Josef-Straße 18, A-8700 Leoben, Austria; neda.abdoshahi@unileoben.ac.at (N.A.); svea.mayer@unileoben.ac.at (S.M.); helmut.clemens@unileoben.ac.at (H.C.)

**Keywords:** thermal expansion, titanium aluminides, thermodynamic properties, ab initio calculations, quasi-harmonic approximation

## Abstract

Anisotropic thermal expansion coefficients of tetragonal γ-TiAl and hexagonal $\alpha_{2}$-Ti_3_Al phases were calculated using first principles methods. Two approaches with different computational costs and degrees of freedom were proposed. The predicted values were compared with available experimental data showing that for γ-TiAl, the more computational demanding method with decoupled impact of volume and temperature effects on the cell shape leads to significantly better results than that with only ground-state optimised unit cell geometry. In the case of the $\alpha_{2}$-Ti_3_Al phase, both approaches yielded comparable results. Additionally, heat capacity and bulk modulus were evaluated as functions of temperature for both phases, and were fitted to provide an analytical formula which can be further used.

## 1. Introduction

First principles calculations are now a widely used and well-established method for complementing experimental materials science research [1]. Despite the fact that many recent activities have been directed towards big-data and machine learning [2,3,4], there are still many topics which require individualised treatments. An example of such a problem is the discrepancy between the experimentally and theoretically reported stability and chemistry of the Nb_3_Al phase also published in this special issue [5]. Starting from the pioneering works of Grabowski and co-workers [6,7,8,9,10,11], the first-principles thermodynamics by including the vibrational contribution to the free energy within the harmonic approximation have became fairly routine. Among other available tools, phonopy [12] has become widely used thanks to its robustness, openness and flexibility. The there implemented quasi-harmonic approximation (QHA) for calculating thermal properties, such as thermal expansion, bulk modulus or heat capacity, however, does not include effects of temperature-induced changes in the unit cell geometry in terms of c/a or b/a ratios or lattice angles, as may be the case of systems with lower than cubic symmetry.

In this paper we will focus on γ-titanium aluminides, which are a class of intermetallic materials with a broad range of potential high temperature applications [13,14,15,16,17]. They exhibit good specific yield strength and elastic moduli at elevated temperatures, while simultaneously having low density, good oxidation resistance and resistance against Ti-fire [18,19,20,21,22]. Depending on the exact chemical composition, several phases are present in TiAl alloys [23]. The majority phase is the tetragonal γ-TiAl phase (tetragonal L$1_{0}$, space group P4/mmm) after which this material class is named. In addition, the $\alpha_{2}$-Ti_3_Al phase (hexagonal D$0_{19}$, space group P6_3_/mmc) is also present in alloys of industrial relevance. When the solidification proceeds via the β-phase field, the ordered $\beta_{o}$-TiAl phase (B2, Pm$\bar{3}$m) may also be detected at room temperature (RT) [24,25,26]. A careful selection of the alloy processing route, by which the phase volume fractions and grain morphology are adjusted, results in optimising the TiAl mechanical properties within certain limits [14,27,28]. The $\beta_{o}$ phase, however, does not appear in the pure binary Ti–Al system [29], and therefore will not be discussed here anymore.

Many material parameters are needed as inputs for the precise consideration of structural materials. Thermodynamic data such as heat capacities and Gibbs free energies are essential inputs for Calphad-based modelling, as demonstrated, e.g., by a recent reassessment of the Ti–Al–Mo ternary system [30]. Nevertheless, for structural materials for high temperature applications, as is the case of TiAl-based alloys, other properties are equally important for predicting a precise microstructural state including internal stresses. Among these is coefficient of thermal expansion (TEC), α, which is not part of a standard thermodynamic assessment. This is demonstrated, for example, in Refs. Nabarro [31], Schuh et al. [32], where the influence of the anisotropic thermal expansion in γ-TiAl on the creep behaviour under cyclic thermal loading is discussed. Further on, the authors suggested that the effect of thermal cycling is expected to be significantly higher in the $\alpha_{2}$-phase and that ratcheting creep is to be expected in polycrystalline specimens. However, due to the lack of thermal expansion data, this postulation could not be substantiated.

Several techniques have been used to estimate TEC experimentally. Zupan and Hemker [33] used micro tensile testing to study γ single crystals. He et al. [34] presented a comprehensive investigation of γ-Ti_44_Al_56_ (Ti-56Al, in atomic percent) using a capacitance dilatometer to determine TEC along the *a* (γ-[100]) and *c* (γ-[001]) directions in the temperature range between 0 and 750K. Bittorf et al. [35] determined the TEC of γ single-phase polycrystalline specimen of Ti_45_Al_54_ (Ti-54Al in atomic percent) by means of neutron diffraction, which are very close to those of He et al. [34]. Both these studies suggest that $\alpha_{a}>\alpha_{c}$.

Thermal expansion of polycrystalline multiphase specimens was investigated by Stone and Kurfess [36] and Zhang et al. [37] employing dilatometric techniques. In these cases, however, the results describe the overall thermal expansion behaviour of the investigated alloys, and do not allow for distinguishing TEC of individual phases. Novoselova et al. [38] utilised high-energy X-ray diffraction (HEXRD) to determine various material parameters including TEC, for the $\alpha_{2}$ and γ-phase in a polycrystalline specimen of Ti–46Al–1.9Cr–3Nb (at. %), in the temperature range from 0–$1500{\;}^{\circ }C$. Unfortunately, this study provides only a low number of data points between 0 and $1000{\;}^{\circ }C$. Recently, Li et al. [39] published a study on Ti-45Al-7.5Nb-0.5C (at. %), in which they report on thermal strains in individual (γ and $\alpha_{2}$) phases using synchrotron diffraction. They also suggest that $\alpha_{a}$ is slightly larger than $\alpha_{c}$, although they do not focus on the low-temperature regime below 1000K and report only a single value independent of temperature (linear thermal expansion with respect to room temperature lattice constants). In contrast to Refs. [34,35,39], Novoselova et al. [38] obtained $\alpha_{a}$ ≈ 2.5-times smaller than $\alpha_{c}$. It is therefore reasonable to expect that TEC is strongly composition-dependent.

Other phases present in the Ti–Al system, in particular the $\alpha_{2}$-Ti_3_Al phase, have received very little attention and data on their thermal expansion coefficients are scarce. While Novoselova et al. [38] reported $\alpha_{a}>\alpha_{c}$ for Ti-46Al-1.9Cr-3Nb (at. %), Li et al. [39] measured TEC for Ti-45Al-7.5Nb-0.5C (at. %) in both directions essentially the same and more than twice higher than in the formed case. Despite both these studies are not for pure phases, we can conclude that in this case TEC is also likely to be strongly composition-dependent.

As a counterpart to the experimentally estimated values of TEC, first principles quantum mechanical calculations were used by Fu et al. [40] to predict the thermal expansion behaviour of the γ-TiAl phase at pressures ranging from 0 (ambient pressure) to 100GPa. However, in comparison with the experimental TEC obtained by He et al. [34], the calculated values are significantly higher. Moreover, the authors did not account for the tetragonality of the γ-TiAl, i.e., the possible anisotropy of TEC.

Therefore, in the current work, we employ first principles calculations within the quasi-harmonic approximation to reveal TEC of the $\alpha_{2}$-Ti_3_Al and γ-TiAl phases with a special focus on determining the anisotropy of this property.

## 2. Methods

We used the state-of-the-art program VASP (Vienna Ab-initio Simulation Package) [41] employing Density Functional Theory [42,43] to carry out the first principles calculations. The atomic basis functions were represented by projector augmented wave pseudopotentials with the 3$s^{2}$3$p^{6}$4$s^{2}$3$d^{2}$ and 3$s^{2}$2$p^{1}$ valence electron configuration for Ti and Al atoms, respectively. The exchange-correlation effects were treated using gradient corrected exchange-correlation functional parametrised by Perdew–Burke–Ernzerhof (GGA-PBE) [44,45] and the plane wave cut-off energy of 500eV were applied to predict ground state properties of both the γ-TiAl and $\alpha_{2}$-Ti_3_Al phases. The reciprocal unit cell was sampled with 14×14×14 (γ, 4 atoms) and 12×12×13 ($\alpha_{2}$, 8 atoms) **k**-point mesh using the Monkhorst–Pack scheme. These parameters guarantee total energy accuracy better than 1meV/at.

The structural optimisation includes evaluation of total energies at various volumes. Full relaxation including unit cell shape and internal atomic coordinates optimisation was performed for every volume, yielding lattice parameters $a_{0}^{\xi }\left(V\right)$ and $c_{0}^{\xi }\left(V\right)$ (ξ=γ or $\alpha_{2}$) as functions of volume at 0K.

Thermal properties were evaluated within the quasi-harmonic approximation (QHA) using the phonopy code [12,46]. The phonon frequencies were calculated for 6 evenly spaced volumes in the range 15.4–$17.4\;{Å}^{3}/\mathrm{at}.$ (γ-phase) and 6 volumes in the range 15.8–$17.9\;{Å}^{3}/\mathrm{at}.$ ($\alpha_{2}$-phase) employing 3×3×3 (54 atoms) and 2×2×2 (64 atoms) supercells, respectively.

Assuming that the c/a ratio is only a function of volume and not temperature, the resulting temperature dependence of volume $V^{\xi }\left(T\right)$ as obtained from the QHA (phonopy-qha package), allows to determine also the temperature dependencies of individual lattice constants $x^{\xi }\left(T\right)$, x=a,c and $\xi =\gamma ,\alpha_{2}$, as:

(1) $$x^{\xi }\left(T\right)=x_{0}^{\xi }\left(V\left(T\right)\right)\;.$$

This treatment is in the following termed as ’ground state optimised cell shape’ (gs-cs).

To probe the validity of the assumption that the c/a is only a function of volume independent of temperature, we have adopted additional scheme. For every volume, we selected 5 c/a ratios around the GGA-PBE equilibrium values (${\left(c/a\right)}_{\gamma }^{\mathrm{GGA}-\mathrm{PBE}}=1.018$, ${\left(c/a\right)}_{\alpha_{2}}^{\mathrm{GGA}-\mathrm{PBE}}=0.809$). For each of these static configurations, thermodynamic properties within the harmonic approximation (phonopy package) were calculated, hence yielding vibrational Helmholtz free energies $F_{\mathrm{vib}}\left(T,V,c/a\right)$. The total Helmholtz free energy, *F*, was constructed by adding the 0K total energies:

(2) $$F\left(T,V,c/a\right)=E_{\mathrm{tot}}\left(V,c/a\right)+F_{\mathrm{vib}}\left(T,V,c/a\right)\;.$$

The equilibrium geometry at a fixed temperature *T* was then calculated by a two step fitting. First, we estimated

(3) $$F\left(T,V\right)=\min_{c/a}F\left(T,V,c/a\right)$$

by fitting the F(T=const.,V=const,c/a) data with a second order polynomial. Subsequently, the F(T,V) data were fitted with the Birch–Murnaghan equation of state [47] to obtain the equilibrium volume $V_{0}\left(T\right)$ (in addition to free energy, F(T), bulk modulus, B(T), and pressure derivative of bulk modulus, $B^{\prime }\left(T\right)$). Finally, the (c/a)(T=const.,V) data minimising F(T=const.,V,c/a) in Equation (3), were linearly interpolated as a function of *V*, and the equilibrium value at temperature *T* was estimated from this linear fit at $V=V_{0}\left(T\right)$. This procedure allows to decouple influence of temperature and pressure (volume) on the cell geometry, and is in the following thus termed ’temperature optimised cell shape’ (to-cs) approach.

The thermal expansion coefficients were calculated from the estimated lattice parameters as

(4) $$\alpha_{x}^{\xi }\left(T\right)=\frac{1}{x_{\xi }\left(T\right)}\frac{dx_{\xi }}{dT}\approx \frac{1}{x_{\xi }\left(T\right)}\frac{x_{\xi }\left(T+\Delta T\right)-x_{\xi }\left(T-\Delta T\right)}{2\Delta T}\;.$$

Finally, the heat capacity at constant (ambient) pressure, $C_{p}$, was estimated from the Helmholtz free energy, $F^{\xi }\left(T\right)$, as

(5) $$C_{p}\left(T\right)=-T\frac{\partial^{2}F\left(T\right)}{\partial T^{2}}\approx -T\frac{F(T+\Delta T)+F(T-\Delta T)-2F(T)}{{\left(\Delta T\right)}^{2}}\;.$$

The latter expressions in Equations (4) and (5) represent numerical derivatives as both, lattice constants and Helmholtz free energy were calculated on a discrete set of temperatures from 0 to 1000K with a step of 10K.

## 3. Results

### 3.1. Thermal Expansion

**Figure 1:**
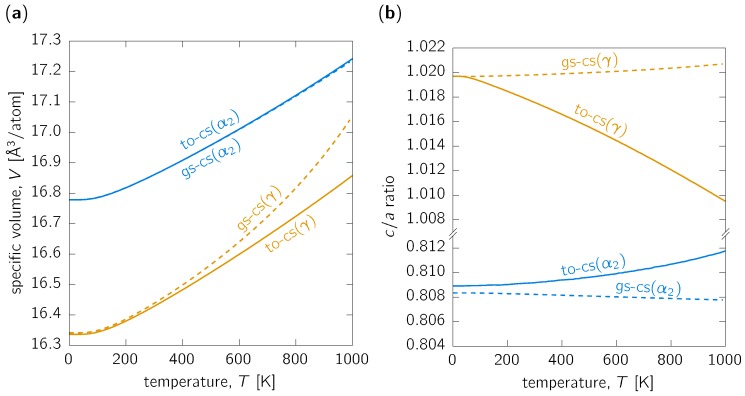
(**a**) Specific volume and (**b**) c/a ratio as functions of temperature for the $\alpha_{2}$-Ti_3_Al (blue) and γ-TiAl (orange) phases predicted using quasi-harmonic approximation with cell shape optimised at 0K (dashed, label ‘gs-cs’ (ground state optimised cell shape)) and at every temperature (solid line, label ‘to-cs’ (temperature optimised cell shape)).

We start our analysis by comparing the predicted temperature dependence of specific volumes of the $\alpha_{2}$-Ti_3_Al and γ-TiAl phases using both approaches as described in the Section 2. Figure 1a shows the temperature dependence of specific volume (i.e., volume per atom) for both considered phases as predicted using volume geometries optimised only at 0K (gs-cs) and at every temperature (to-cs). While these two approaches provide almost identical results for the $\alpha_{2}$-Ti_3_Al phase (blue curves), significant differences are obtained for the γ-TiAl. Namely, the gs-cs method yields larger and faster expanding volumes than the to-cs treatment. The former is also significantly non-linear, suggesting that the resulting coefficient of volume thermal expansion is strongly increasing at higher temperatures and does not reach the usual near-to-linear behaviour for temperatures above room temperature (RT, ∼298K).

Importantly, both approaches allow for explicitly estimating *a* and *c* lattice constants describing the hexagonal $\alpha_{2}$-Ti_3_Al and tetragonal γ-TiAl structures. Similarly to the specific volume, the c/a ratio for the $\alpha_{2}$ structure also does not differ much for both the gs-cs and to-cs approaches. While the absolute values do not differ very much, the qualitative temperature-dependence changes from c/a decreasing with temperature as predicted by the gs-cs method to c/a increasing with raising temperature for the to-cs approach (see Figure 1b). Qualitatively similar behaviour is also predicted for the γ-TiAl phase, although in the opposite sense: the gs-cs and to-cs methods predict slightly increasing and strongly decreasing c/a values, respectively, with increasing temperature.

**Figure 2:**
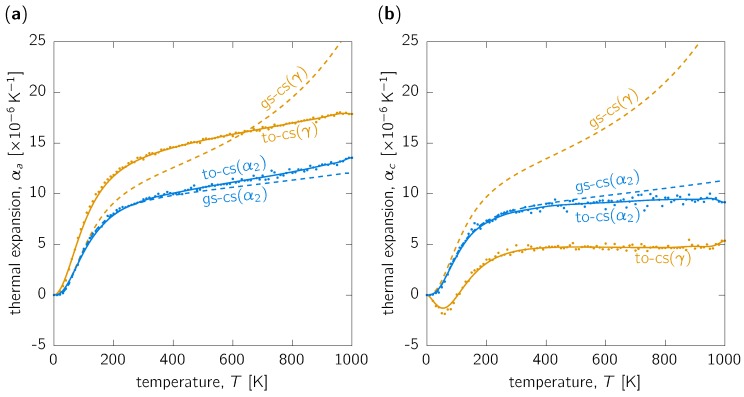
Lattice thermal expansion along (**a**) *a*-direction, $\alpha_{a}$, and (**b**) *c*-direction, $\alpha_{c}$, as functions of temperature for the $\alpha_{2}$-Ti_3_Al (blue) and γ-TiAl (orange) phases predicted using quasi-harmonic approximation with cell shape optimised at 0K (dashed, label ‘gs-cs’) and at every temperature (solid line, label ‘to-cs’). The data points shown by dots are the actual numerically calculated values using Equation (4). The smooth curves are ‘guides for the eyes’ from interpolation using Bezier curves.

The specific volume and c/a ratio allow to calculate also the corresponding lattice parameters *a* and *c*, and then to further use these to obtain lattice thermal expansion coefficients, $\alpha_{a}$ (Figure 2a) and $\alpha_{c}$ (Figure 2b), according to Equation (4). Regarding the hexagonal $\alpha_{2}$-Ti_3_Al phase, $\alpha_{a}$ is slightly larger than $\alpha_{c}$ for all temperatures. Perhaps the most important difference is that while $\alpha_{a}$ still increases with temperature even above RT and to higher values than $10\times 10^{-6}\;K^{-1}$ above ∼600K, while $\alpha_{c}$ seems to quickly saturate around $10\times 10^{-6}\;K^{-1}$ above RT. Importantly, there are no significant differences between the predicted values by gs-cs and to-cs methods. The obtained differences are of the same order as the scatter of the numerical noise imposed by the to-cs method, represented by the individual data points in Figure 2.

A very different situation is obtained in the γ-TiAl case. The gs-cs case predicts significant temperature dependence of both $\alpha_{a}$ and $\alpha_{c}$, moreover, both having very similar values. This large increase of TEC with temperature is a consequence of strongly expanding volume of the γ-TiAl (cf. Figure 1a) resulting from the gs-cs method. On the other hand, the to-cs approach predicts large TEC values of ∼15 ×$10^{-6}\;K^{-1}$ above RT in the *a*-direction, while 3-fold smaller values of around $5\times 10^{-6}\;K^{-1}$ (and basically temperature-independent above RT) are predicted for $\alpha_{c}$. This behaviour leads to a strong temperature dependence of c/a (cf. Figure 1b).

In summary, while the computationally more demanding to-cs method does not yield too different temperature dependence of structural properties in comparison to the simpler gs-cs approach for the $\alpha_{2}$-Ti_3_Al, non-negligible differences are obtained in the case of the γ-TiAl.

### 3.2. Other Thermodynamic Properties

The calculation of the thermal expansion is based on evaluation of the vibrational entropy term of the Helmholtz free energy, which is the most important contribution, and has been demonstrated several times to be the only important contribution when dealing with non-magnetic materials at temperatures far below melting point [9,11]. The thus obtained thermodynamic potentials, however, offer thermal dependence of other quantities, too.

**Figure 3:**
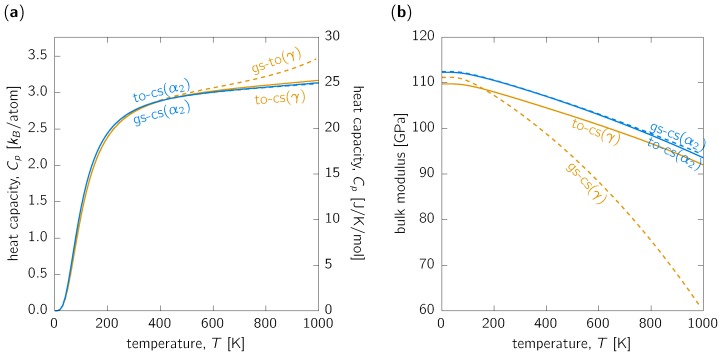
(**a**) Heat capacity at constant (ambient) pressure and (**b**) bulk modulus of the $\alpha_{2}$ (blue lines) and γ (orange lines) phase as functions of temperature evaluated within the gs-cs (dashed lines) and to-cs (solid lines) approach.

The heat capacity, $C_{p}$, at constant (ambient) pressure was evaluated according to Equation (5). The calculated values for the two phases are almost identical, in particular from the to-cs treatment (Figure 3a). This result could be intuitively understood by the fact that the molar heat capacities of Al and Ti are very similar [6]. Such prediction is important, e.g., for the discussion of microstructure evolution upon phase transformations during cooling.

The temperature dependence of bulk modulus (i.e., the inverse of compressibility) (Figure 3b) can be estimated from fitting section of the Helmholtz free energy surface at fixed temperature with, e.g., the Birch–Murnaghan equation of state [47]. It turns out that the bulk modulus of the γ-phase is smaller than that of the $\alpha_{2}$-phase in the whole temperature range up to 1000K. The bulk modulus softens with the increasing temperature by ∼12% between 0K and 770K (∼500${\;}^{\circ }C$) and by approximately ∼8.5% between RT and $500{\;}^{\circ }C$ for both, γ and $\alpha_{2}$-phases. Significantly different is only the gs-cs temperature dependence for the γ-phase, which yields drop of over 30% between 0 and 770K, further underlying that this approach is not reasonable for the γ-TiAl, in accordance with other properties discussed so far.

In order to provide the reader with an easy access to our calculated quantities, the trends were fitted with analytical expressions and the resulting fitted parameters as well as the fits themselves are summarised in Appendix A.

### 3.3. Discussion

As mentioned in the introduction, experimental data for comparison are scarce. In fact, data corresponding to exactly ideal conditions of single phases with exact stoichiometric compositions are non-existant at all. Nevertheless, experimental data on single crystal [34], as well as on polycrystalline γ-TiAl [35], suggest that $\alpha_{a}>\alpha_{c}$, in agreement with our calculations. On the one hand, the differences between *a* ([100]) and *c* ([001]) directions are not so large in experiments as they are predicted here (cf. Figure 2), on the other hand the experimental data are for Al-rich compositions with 54 and 56 at.% Al. That the composition can play a significant role is demonstrated by the hugely different TEC reported for Ti-46Al-3Nb-1.9Cr (at. %) [38] and Ti-45Al-7.5Nb-0.5C (at. %) [39]. In the light of these hugely scattering experimental data, our predictions are qualitatively correct in implying $\alpha_{a}>\alpha_{c}$.

The structurally optimised c/a ratio of ∼1.020 is in excellent agreement with the experimentally reported values 1.016 [48] for Al-rich γ-TiAl to 1.02 [17]. The latter two values are higher than the ∼1.012 measured for Ti-45Al-7.5Nb-0.5C (at. %) [39]. Importantly, Li et al. [39] obtained a slightly decreasing c/a ratio with increasing temperature up to 1000K (followed by a strong decrease for temperatures further increasing up to 1400K), a result qualitatively well in agreement with our to-cs predictions. Further on, Li et al. [39] reported that the c/a of $\alpha_{2}$-Ti_3_Al stays rather constant, i.e., ∼0.806, in the temperature range from 450 to 1000K. Despite the fact that the gs-cs and to-cs approaches yield decreasing and increasing c/a with temperature, respectively, the temperature dependence is not very strong (as, e.g., in the case of the γ-TiAl phase) and hence both methods are, in fact, valid for the $\alpha_{2}$-phase. It should also be mentioned that experimental results on polycrystalline specimens may be biased by building up coherency strains between phases with different expansion behaviour [49]. Especially in the case of TiAl alloys, which contain a large volume fraction of lamellar $\alpha_{2}$/γ colonies, this effect may potentially have a significant impact on the obtained experimental data.

Finally, our calculated values of TEC agree with experimental results, but are significantly lower than other DFT-based predictions by Fu et al. [40]. We ascribe this discrepancy to the different methodology used: in the present study, we have explicitly evaluated the vibrational contribution to the Helmholtz free energy by calculating phonon properties, whereas Fu et al. [40] used a semi-classical Debye model. We therefore conclude that explicit evaluation of the phonon frequencies and their contribution to the phonon free energy is essential.

## 4. Conclusions

Thermal properties, with a special focus on structural analysis of temperature dependent lattice parameters and coefficients of thermal expansion of tetragonal γ-TiAl and hexagonal $\alpha_{2}$-Ti_3_Al phases of binary Ti–Al system, were calculated using first principles methods. We put our attention on testing whether the c/a ratio is purely a function of volume independent of temperature, or whether temperature and volumetric effects have to be separated. Our calculations show that in the case of the γ-TiAl phase significant differences are obtained, while both approaches yield comparable results for the hexagonal $\alpha_{2}$-Ti_3_Al phase. The predictions were further compared with available experimental data. While this was not straightforward due to lack of single-crystalline data with close-to-perfect stoichiometries, we propose that the to-cs method with decoupled impact of temperature and volume on the cell geometry (c/a ratio) gives better agreement for the γ-TiAl phase. The present paper therefore contributes to advancing first principles thermodynamics beyond systems with cubic symmetry.

## Figures and Tables

**Figure A1:**
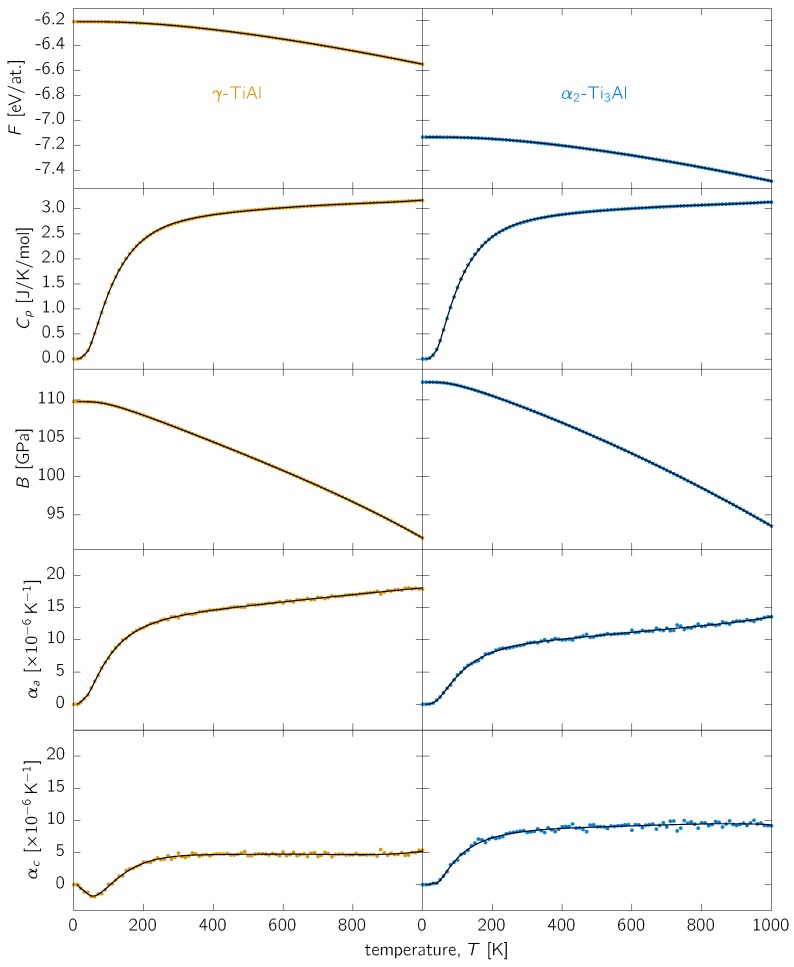
Fits of temperature optimised cell shape (to-cs) calculated thermodynamic properties using Equation (A1) and coefficients from Table A1 (γ-TiAl, left column) and Table A2 ($\alpha_{2}$-Ti_3_Al, right column). The actual calculated data are shown by the small coloured points while the fits are thin continuous black lines.

## Abbreviations

The following abbreviations are used in this manuscript:

DFT	Density Functional Theory
GGA	Generalised Gradient Approximation
gs-cs	ground state optimised cell shape
HA	harmonic approximation
QHA	quasi-harmonic approximation
RT	room temperature
TEC	coefficient of thermal expansion
to-cs	temperature optimised cell shape

## Appendix A. Analytical Fits

The thermodynamic quantities discussed above calculated using the to-cs approach were fitted with a function of the form

(A1) $$X\left(T\right)=a_{0}^{X}+\sum_{i=1}^{4}a_{i}^{X}T^{i}+\sum_{i=1}^{4}b_{i}^{X}\frac{1}{T^{i}}+c^{X}\ln\left(T\right)\;.$$

This function fits accurately all obtained data within the temperature window from 0 to 1000K. The fitted coefficients for X=F (Helmholtz free energy), $C_{p}$ (molar heat capacity), *B* (bulk modulus) and $\alpha_{a}$ and $\alpha_{c}$ (TEC in the *a* and *c* directions) are summarised in Table A1 and Table A2, and the fits are presented in Figure A1. The calculated dependencies provide a consistent set of material constants.

**Table A1:** Fitted coefficients according to Equation (A1) for the calculated thermodynamic properties of the γ-TiAl phase. *F* is Helmholtz free energy [eV/at.], $C_{p}$ is molar heat capacity at constant pressure [J/K/mol] (mol of atoms), *B* is bulk modulus [GPa] and $\alpha_{a}$ and $\alpha_{c}$ are coefficient of thermal expansion (TEC) in the [100] and in the [001] directions [$K^{-1}$].

	*F*	$C_{p}$	*B*	$\alpha_{a}$	$\alpha_{c}$
	[eV/at.]	[J/K/mol]	[GPa]	[$K^{-1}$]	[$K^{-1}$]
$a_{0}$	$-6.4187\times 10^{+00}$	$-3.4558\times 10^{+01}$	$1.1499\times 10^{+02}$	$-3.6457\times 10^{-05}$	$-3.8562\times 10^{-04}$
$a_{1}$	$-1.8683\times 10^{-04}$	$-4.2978\times 10^{-02}$	$-1.8487\times 10^{-02}$	$-7.0845\times 10^{-08}$	$-3.9878\times 10^{-07}$
$a_{2}$	$-3.7211\times 10^{-07}$	$5.0198\times 10^{-05}$	$1.5144\times 10^{-06}$	$8.5104\times 10^{-11}$	$4.5801\times 10^{-10}$
$a_{3}$	$1.5691\times 10^{-10}$	$-3.5403\times 10^{-08}$	$-6.3023\times 10^{-10}$	$-5.4782\times 10^{-14}$	$-3.2999\times 10^{-13}$
$a_{4}$	$-3.7138\times 10^{-14}$	$1.0447\times 10^{-11}$	$-2.0214\times 10^{-12}$	$1.4449\times 10^{-17}$	$1.0165\times 10^{-16}$
$b_{1}$	$3.4305\times 10^{+00}$	$2.9671\times 10^{+02}$	$-2.1317\times 10^{+02}$	$-7.2401\times 10^{-04}$	$6.0522\times 10^{-03}$
$b_{2}$	$-6.0577\times 10^{+01}$	$-2.0669\times 10^{+01}$	$5.5554\times 10^{+03}$	$3.6802\times 10^{-02}$	$-9.7074\times 10^{-02}$
$b_{3}$	$6.3758\times 10^{+02}$	$-3.9874\times 10^{+04}$	$-6.9252\times 10^{+04}$	$-5.5989\times 10^{-01}$	$9.1203\times 10^{-01}$
$b_{4}$	$-2.6280\times 10^{+03}$	$2.7003\times 10^{+05}$	$3.1027\times 10^{+05}$	$2.7435\times 10^{+00}$	$-3.4160\times 10^{+00}$
*c*	$4.4277\times 10^{-02}$	$7.9870\times 10^{+00}$	$-4.5702\times 10^{-01}$	$1.1764\times 10^{-05}$	$8.0199\times 10^{-05}$

**Table A2:** Fitted coefficients according to Equation (A1) for the calculated thermodynamic properties of the $\alpha_{2}$-Ti_3_Al phase. *F* is Helmholtz free energy [eV/at.], $C_{p}$ is molar heat capacity at constant pressure [J/K/mol] (mol of atoms), *B* is bulk modulus [GPa] and $\alpha_{a}$ and $\alpha_{c}$ are TEC in the (0001) plane and in the [0001] direction [$K^{-1}$].

	*F*	$C_{p}$	*B*	$\alpha_{a}$	$\alpha_{c}$
	[eV/at.]	[J/K/mol]	[GPa]	[$K^{-1}$]	[$K^{-1}$]
$a_{0}$	$-7.3540\times 10^{+00}$	$-2.0371\times 10^{+01}$	$1.1716\times 10^{+02}$	$-1.6008\times 10^{-04}$	$5.4128\times 10^{-05}$
$a_{1}$	$-2.1062\times 10^{-04}$	$-3.1196\times 10^{-02}$	$-1.4167\times 10^{-02}$	$-1.8981\times 10^{-07}$	$2.5208\times 10^{-08}$
$a_{2}$	$-3.6153\times 10^{-07}$	$3.8017\times 10^{-05}$	$-4.9187\times 10^{-06}$	$2.3150\times 10^{-10}$	$-3.7248\times 10^{-11}$
$a_{3}$	$1.5105\times 10^{-10}$	$-2.7416\times 10^{-08}$	$-6.5580\times 10^{-10}$	$-1.6951\times 10^{-13}$	$3.9920\times 10^{-14}$
$a_{4}$	$-3.5505\times 10^{-14}$	$8.2107\times 10^{-12}$	$6.6516\times 10^{-14}$	$5.2923\times 10^{-17}$	$-1.6988\times 10^{-17}$
$b_{1}$	$3.4451\times 10^{+00}$	$1.9711\times 10^{+01}$	$-1.6377\times 10^{+02}$	$1.8767\times 10^{-03}$	$-2.2451\times 10^{-03}$
$b_{2}$	$-5.9472\times 10^{+01}$	$5.5440\times 10^{+03}$	$3.9202\times 10^{+03}$	$-1.9966\times 10^{-02}$	$6.8760\times 10^{-02}$
$b_{3}$	$6.1738\times 10^{+02}$	$-1.0178\times 10^{+05}$	$-4.6560\times 10^{+04}$	$1.1511\times 10^{-01}$	$-9.5646\times 10^{-01}$
$b_{4}$	$-2.5244\times 10^{+03}$	$5.3097\times 10^{+05}$	$2.0274\times 10^{+05}$	$-2.3378\times 10^{-01}$	$4.5685\times 10^{+00}$
*c*	$4.6143\times 10^{-02}$	$5.1928\times 10^{+00}$	$-5.4515\times 10^{-01}$	$3.5711\times 10^{-05}$	$-7.7496\times 10^{-06}$
